# Adhesion mechanisms mediated by probiotics and prebiotics and their potential impact on human health

**DOI:** 10.1007/s00253-019-09978-7

**Published:** 2019-07-02

**Authors:** Andrea Monteagudo-Mera, Robert A. Rastall, Glenn R. Gibson, Dimitris Charalampopoulos, Afroditi Chatzifragkou

**Affiliations:** 1 Biomedical Sciences, School of Biological Sciences, University of Reading, Reading, RG6 6AH UK; 2 Department of Food and Nutritional Sciences, University of Reading, Whiteknights, PO Box 226, Reading, RG6 6AP UK

**Keywords:** Probiotics, Prebiotics, Adhesion, Pathogens, Immunomodulation

## Abstract

Adhesion ability to the host is a classical selection criterion for potential probiotic bacteria that could result in a transient colonisation that would help to promote immunomodulatory effects, as well as stimulate gut barrier and metabolic functions. In addition, probiotic bacteria have a potential protective role against enteropathogens through different mechanisms including production of antimicrobial compounds, reduction of pathogenic bacterial adhesion and competition for host cell binding sites. The competitive exclusion by probiotic bacteria has a beneficial effect not only on the gut but also in the urogenital tract and oral cavity. On the other hand, prebiotics may also act as barriers to pathogens and toxins by preventing their adhesion to epithelial receptors. In vitro studies with different intestinal cell lines have been widely used along the last decades to assess the adherence ability of probiotic bacteria and pathogen antagonism. However, extrapolation of these results to in vivo conditions still remains unclear, leading to the need of optimisation of more complex in vitro approaches that include interaction with the resident microbiota to address the current limitations. The aim of this mini review is to provide a comprehensive overview on the potential effect of the adhesive properties of probiotics and prebiotics on the host by focusing on the most recent findings related with adhesion and immunomodulatory and antipathogenic effect on human health.

## Introduction

Numerous studies carried out during the last two decades have evidenced the importance of the gut microbiota on health and disease. Dysbiosis in the structure of the gut microbial community has been associated with multiple intestinal diseases and metabolic disorders such as inflammatory bowel diseases (IBDs), diabetes and obesity (Musso et al. 2011; Wlodarska et al. 2015). In this regard, the role of probiotics and prebiotics as modulators of the microbiota has being widely investigated for the treatment and prevention of diseases (Markowiak and Ślizewska 2017).

Probiotics are defined as “live microorganisms that, when administered in adequate amounts, confer a health benefit on the host” (Hill et al. 2014) whilst a prebiotic is “a substrate that is selectively utilized by host microorganisms conferring a health benefit” (Gibson et al. 2017). On the other hand, prebiotics are non-viable substrates that serve as nutrients for beneficial microorganisms harboured by the host, including administered probiotic strains and resident microorganisms (Gibson et al. 2017). Within the latest definition of a prebiotic, it is expected that it could invoke changes to any host microbial ecosystem, not just the gut, through their selective utilisation by live host microorganisms.

There are different mechanisms of action through which probiotics and prebiotics can impact human health such as inhibition of pathogenic bacteria, immunomodulatory effects, stimulation of barrier function and metabolic function (Guarner et al. 2012). Although adhesion ability of probiotic bacteria to the host does not necessarily ensure a health benefit, the attachment of probiotics to the intestinal mucosa can have a potential protective role against enteropathogens through competition for host cell binding sites. In addition, adhesion ability of probiotic bacteria could increase the opportunity to interact with the host resulting in a temporary colonisation, increasing their transit time in the gut to exert their intended beneficial effects. For instance, this temporary colonisation favours the local action of metabolites produced by probiotics (as in the case of short-chain fatty acids, SCFA) as well as immunomodulatory effects by bacterial surface-located molecules which act as ligands for host receptors in the intestinal epithelium inducing signalling pathways. On the other hand, oligosaccharides acting as prebiotics could also enhance the adhesion ability of probiotic strains, suggesting that the development of new symbiotic products could be a potential tool to increase the time of residence of probiotic bacteria in the gut (Celebioglu et al. 2017). In addition, prebiotics can also act as decoy receptors, inhibiting the adhesion of some pathogenic bacteria to the intestine as reviewed by Hickey (2012).

The aim of this review is to provide a comprehensive overview on bacterial adhesion, by focusing on the most recent findings that are related with adhesive properties of probiotics, and in some cases of prebiotics, and their potential effect on human health. To this end, insights on the enhancement of gut homeostasis through the transitory effect of probiotics, on the synergistic effects of probiotics and prebiotics to promote the inhibition of pathogen binding to host and their ability to trigger signalling pathways are discussed.

## Adhesion mechanisms of probiotics to intestinal mucosa

Glycocalyx is a layer that contains glycolipids and glycoproteins that covers the intestinal epithelial cells. The viscous consistency of this layer protects the intestinal epithelium from mechanical damage and hinders the colonisation by bacteria protecting the host against bacterial infections. This mucosa layer contains mainly glycosylated proteins (mucins) as well as glycolipids, immunoglobulins and electrolytes (Bron et al. 2012). The sugar residues of mucins can act as ligands for bacterial membrane receptors, and in fact, changes in the glycosylation pattern have been associated to dysbiosis during intestinal inflammation (Larsson et al. 2011; Sommer et al. 2014). Species of *Lactobacillus* and *Bifidobacterium* are the most commonly used probiotic bacteria. Both genera are characterized as Gram-positive lactic acid bacteria and share common surface molecules such as lipoteichoic acid (LTA), surface layer associated proteins (SLAPs) and mucin biding proteins (Mubs) that play an important role in the interaction with mucus components (Lebeer et al. 2010).

Bacterial adhesion to intestinal surfaces could be driven initially by non-specific physical binding as hydrophobic interactions followed by a second stage of adhesion by specific cell wall components (Haddaji et al. 2015). Some researchers have reported a correlation between hydrophobicity and adhesion (Pan et al. 2006). In this regard, the presence of some surface proteins such as cell wall–anchored proteinases has been shown to enhance hydrophobicity and adhesion in some lactic acid bacteria (Muñoz-Provencio et al. 2012; Zhang et al. 2015; Radziwill-Bienkowska et al. 2017). The presence of adhesins in the bacterial cell wall has also an important role in the adhesion of bacteria to the intestine. Mucus-binding proteins are surface adhesive proteins that contain Mub and/or MucBP (MUCin-Binding Protein) domains, able to bind mucins and are linked to the peptidoglycan cell wall by a C-terminal Leu-Pro-any-Thr-Gly motif (LPxTG). Although MucBP domains can be found in different bacterial species, including pathogenic bacteria as in the case of *Listeria monocytogenes* (Popowska et al. 2017), Mub domains are almost exclusively found in lactic acid bacteria isolated from the human gastrointestinal tract (Boekhorst et al. 2006; van Tassell and Miller 2011). Also, fimbriae or pili (thin proteinaceous extensions from bacterial cells) can promote adhesion. Type IV pili have been widely characterized in Gram-negative bacteria. These structures provide bacteria an advantage for colonisation of mucosal surfaces (Hospenthal et al. 2017), but recent studies have shown that Gram-positive bacteria as *Bifidobacterium* also can express this type of pili (O’Connell Motherway et al. 2011; Piepenbrink and Sundberg 2016). In addition, some *Lactobacillus* species can also produce SpaCBA pili (Reunanen et al. 2012; Toh et al. 2013). This type of pili (first recognized and characterized in the probiotic strain *Lactobacillus rhamnosus* LGG) consists of 3 subunits, encoded by the cluster *SpaCBA*, assembled by a sortase. Whilst SpaA is the major fibre component of the pilus, SpaB and SpaC are the minor fibre components. SpaC, located in the tip of the pili, is a mucus binding protein responsible for the high adhesion ability of *Lactobacillus rhamnosus* LGG (Reunanen et al. 2012).

Besides mucus-binding proteins and pili, other surface proteins like fibronectin-binding proteins (FBPs) and surface-layer proteins (SLPs) can contribute to the adherence of bacteria to the intestinal mucosa. Fibronectin is an extracellular matrix glycoprotein that can be found in an insoluble form in the intestine. FBPs have been characterized both in Gram-negative and Gram-positive bacteria. The presence of these proteins has been associated with virulence of some pathogens, due to its potential to invade the host epithelial cells. However, the presence of FBPs could be beneficial on probiotic bacteria as they could increase their adhesion ability to host cells favouring the exclusion of pathogens (Lehri et al. 2015; Hymes et al. 2016). On the other hand, SLPs are extracellular para-crystalline proteins that cover the cell surface of bacteria and possess different roles such as structural components, virulence in pathogenic bacteria, antifouling coating or adhesion promoters (Sleytr et al. 2014). The distribution and type of SLPs vary among strains, but these proteins seem to be essential in the adhesion of probiotic bacteria to intestinal cells, as it has been reported a reduction of adhesion after SLPs removal by chemical treatments (Tallon et al. 2007; Zhang et al. 2013). In addition, SLPs could produce immunological response by interaction with host intestinal receptors having as well a role as immunomodulator factor in probiotic bacteria (Konstantinov et al. 2008).

Adherence of probiotic bacteria has been commonly evaluated in vitro using mucin adsorbed onto abiotic surfaces and human tumorigenic cell lines such as Caco-2 and HT-29 (Lebeer et al. 2012; Monteagudo-Mera et al. 2012; Tuo et al. 2013; Garriga et al. 2015) to mimic the adhesion to intestinal epithelial cells (IECs). The use of epithelial cell lines has been extremely useful for the identification of adhesion mechanism and molecules. For example, Wang and colleagues (Wang et al. 2017) using the cell line HT-29 recently identified a new surface layer protein (choline-binding protein A) essential for the adherence of the novel probiotic strain *Lactobacillus salivarius* REN. The identification of adhesive molecules and their genes could be useful for the creation of vectors to increase the adhesive efficiency of other probiotic strains (Hsueh et al. 2010; Zhang et al. 2015). Specifically, the low adhesive ability of the probiotic strain *L. casei* ATCC 393 was increased when a collagen binding protein gen was cloned from the probiotic *L. reuteri* Pg4, leading also to higher competition ability against pathogens in Caco-2 cells (Hsueh et al. 2010). In addition, in vitro studies using cell lines have been useful to predict the effect of probiotics and the gastrointestinal conditions on the adhesive ability of probiotic strains (Deepika et al. 2012; Nivoliez et al. 2014).

Although in vitro experiments are key to understand the mechanisms of adhesion and select probiotic candidates with potential to adhere in vivo, it is difficult to extrapolate these results to the human intestinal tract where other factors such as peristaltic movements, host defence system or competition with resident microbiota could interfere in the attachment. In addition, sugar composition on the cell surface of these tumorigenic cell lines is different to normal intestinal epithelial cells found in the gut (Park et al. 2015) which could not provide us with the real picture about the type of interactions with bacteria. For these reasons, the use of other in vitro models as 3D co-cultured systems integrating the intestinal epithelium and immune cells could be more appropriate to study in more detail the host bacteria interactions and the immune responses caused by probiotics or bacterial infection (Trapecar et al. 2014; Noel et al. 2017).

On the other hand, in vivo and ex vivo studies are more scarce (Dunne et al. 2004; Larsen et al. 2009). In human trials, probiotic strains are usually determined in stool samples due to the non-invasiveness of the technique (Mai et al. 2017). Although the recovery of probiotic bacteria from faeces is an indication of bacterial survival from harsh conditions in the gastrointestinal tract, it is not indicative of the number of total bacteria adhered to the intestinal mucosa. In this regard, studies with more invasive protocols (e.g. biopsies) could enlighten the underpinning mechanisms on adherence of probiotic in the colon. In a human study conducted by Zmora et al. (2018) with healthy volunteers, a multistrain probiotic preparation was administered bi-daily for 4 weeks. Besides stool samples, deep endoscopy and colonoscopy were performed to collect samples from the upper and lower gastrointestinal tract (UGT and LGT). Results showed that 9 out of 11 probiotic strains were significantly detected in the mucosa (LGT) of the supplemented group compared to baseline. However, the authors observed marked inter-individual colonisation patterns which were indistinguishable by probiotic detection in the stool samples. These findings highlight the role of the indigenous microbiota on the adherence of probiotic bacteria and that the development of in vitro models with normal microbiota could provide a more realistic option to assess adhesion properties and mechanisms of probiotic strains. Although germ free/gnotobiotic animals are a good alternative to study probiotic action in the gut environment, host genetics and translation to humans is still a limitation. Novel technologies as gut-on-a-chip models (Jalili-Firoozinezhad et al. 2019) could offer new possibilities in the study of in vitro adherence mechanisms under a microbiota ecosystem with different human intestinal cell populations and peristaltic movements.

## Antagonism against pathogens

### Probiotics

**Fig. 1:**
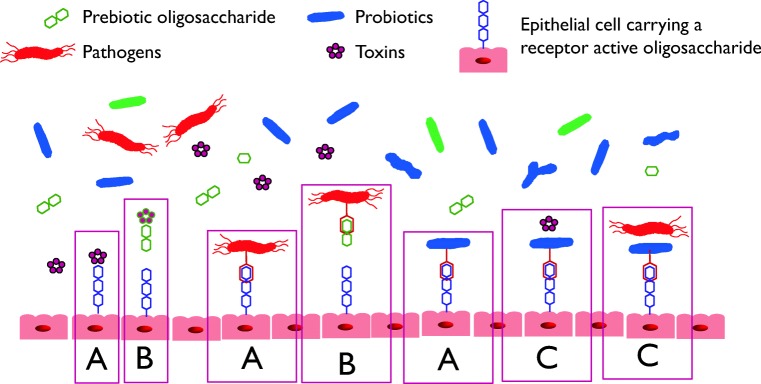
Schematic representation of antiadhesive properties of probiotics and prebiotics. (A) pathogens, probiotic and bacterial toxins adhere to cell surface oligosaccharides carried on glycolipids and glycoproteins; (B) prebiotic oligosaccharides, particularly galacto-oligosaccharides (GOS) have some structural similarity to cell surface glycoproteins and are postulated to inhibit adhesion of toxins and pathogens to cells; (C) the adhesion of probiotic bacteria to cell surface receptors is postulated to inhibit adhesion of toxins and pathogens to those receptors

Antagonism against pathogens is considered as one of the mechanisms of action of probiotic bacteria. This antipathogenic activity is multifactorial including production of antimicrobial compounds and competitive exclusion. Probiotics strains having a good adhesion ability can block the adherence of pathogens by competition for host cell binding sites (Fig. 1). This property is strain specific and it is usually screened in vitro using cell lines infected with pathogens (Walsham et al. 2016; Jessie Lau and Chye 2018; Tuo et al. 2018). For instance, several clinical trials have demonstrated the effectiveness of *Lactobacillus rhamnosus* LGG against vancomycin-resistant enterococci in colonized patients (Szachta et al. 2011; Manley et al. 2007). In this regard, Tytgat et al. (2016) reported a mechanism of competitive exclusion of *Enterococcus faecium* by *L. rhamnosus* GG. In this work, authors demonstrated how the mucus-binding SpaCBA pili of *L*GG, SpaC proteins or antibodies against SpaC inhibited the adhesion of *E. faecium* that possesses similar pili structures.

The competitive exclusion by probiotic bacteria has a beneficial effect not only on the gut but also in other parts of the host where microbiota plays an important role on health, such as the urogenital tract and oral cavity. Numerous evidences have shown that probiotics could be useful for the treatment and prevention of urogenital infections especially in women (Ya et al. 2010; Bisanz et al. 2014; Heczko et al. 2015; Pendharkar et al. 2015). Human urinary bladder epithelium cells are commonly used in vitro to predict the adhesion ability of probiotics and its antagonism against pathogens during urinary infection (Chapman et al. 2014). In this regard, different clinical trials have also reported a transient probiotic colonisation in the vagina and proved the efficacy of probiotic prophylaxis to reduce the incidence of bacterial vaginosis (Verdenelli et al. 2016; Stapleton et al. 2018; Mezzasalma et al. 2017).

Concerning the oral cavity, probiotic administration has been shown to reduce periodontal pathogens both in vitro (Jørgensen et al. 2017) and in vivo studies (Iniesta et al. 2012). For instance, a significant reduction of *Streptococcus mutant* (pathogen related with dental caries) has been observed in several clinical trials during probiotic treatments (Pahumunto et al. 2019; Bafna et al. 2018; Teanpaisan et al. 2015). An increment of probiotic strains in the oral cavity during treatment has been detected in several studies which could indicate a possible transient colonisation of probiotic strains (Krasse et al. 2006; Rungsri et al. 2017). However, different mechanisms of action could be behind of the antagonist effect of probiotic bacteria against pathogens in the oral cavity such as production of antimicrobial compounds, biosurfactant production and adhesion and co-aggregation ability. The use of probiotics in oral health is still controversial, and novel strains isolated from oral cavity must be evaluated to colonize oral niche to avoid affecting the stability of oral communities.

It is important to mention that adhesion ability includes not only the attachment of bacteria cells to host cells but also the attachment to other bacterial cells of different species (co-aggregation) or the same species (auto-aggregation) (Collado et al. 2008). In this regard, multiple studies have shown the potential protective role of probiotics by binding pathogens into co-aggregates inhibiting the biofilm process frequently involved in infections (Matsubara et al. 2016). For instance, a recent in vitro study reported that two probiotic *L. reuteri* strains were able to co-aggregate with *Candida albicans* during biofilm formation creating a hostile environment that inhibited the yeast growth (Jørgensen et al. 2017). Biofilms are defined as a structured community of microorganisms that adheres to surfaces and is enclosed in a self-developed polymeric matrix. Biofilms are frequently associated with infection as the formation of this structure renders pathogens more resistant to host defences and antimicrobial compounds. The formation and development of biofilm by probiotic bacteria can be a beneficial property as it could promote their longer permanency in the intestine, avoiding colonization by enteropathogens. In addition, biofilm-forming probiotic bacteria could be a potential strategy in the control of food-borne pathogens on industrial surfaces. In this regard, bacteriocin LAB producers have been shown to be good candidates to develop protective biofilms to compete and displace pathogenic bacteria (Gómez et al. 2016; Pérez-Ibarreche et al. 2016).

In summary, adhesion to host tissue and antagonism against pathogens by probiotic bacteria are classical screening studies to select probiotic candidates but clinical trials in this field are still scarce and contradictory. Intake of probiotics could lead only to a temporary colonisation, but further improved in vitro and in vivo studies will be required to elucidate the mechanisms and clinical efficacy of probiotics to combat pathogenesis.

### Prebiotics

Apart from the indirect effect of prebiotics as substrates for stimulating the growth of probiotic microorganisms and eventually gut modulation, prebiotics and other non-digestible dietary carbohydrates may offer protection through direct interactions against pathogens (Fig. 1). As previously mentioned, the first step of pathogenesis is the adherence to intestinal epithelial cells, followed by colonisation of the surface in order to produce effective concentrations of toxins that bind to specific surface receptors. Certain oligosaccharides have been reported to exert an antagonistic effect at this stage, by allowing pathogens to bind to the soluble decoy oligosaccharides leading ultimately to displacement or flushing away from the gastrointestinal tract (Shoaf et al. 2006).

Human milk oligosaccharides (HMO) have a structure similar to that of the glycoproteins of the intestinal cells that pathogens target as locations to bind on; this adherence property allows HMO to act as potential infection inhibitors by acting as receptor analogues (Licht et al. 2012). It has been demonstrated in previous studies that the adherence activity of HMO is linked to their fucosylated or sialylated structure (Stahl et al. 1994). Fucosylated and sialylated HMO can protect neonates from infection, by preventing the adhesion of pathogens to the intestinal epithelium. Specifically, human-milk α-1,2-linked fucosylglycans have been shown to inhibit binding by *Campylobacter*, *Vibrio cholera*, stable toxin of *E. coli*, and major strains of caliciviruses in vitro (Sun and Wu 2017). Moreover, high levels of specific 2-linked fucosylglycans in maternal milk were positively correlated with lower risk of diarrhoea from *Campylobacter*, caliciviruses and *E. coli* toxin, and high levels of all 2-linked fucosylglycans in maternal milk were correlated with lower risk of moderate-to-severe diarrhoea in breast-fed infants (Morrow et al. 2004). In the same manner, galacto-oligosaccharides (GOS) have been shown to reduce adherence of enteropathogenic *E. coli* and *Salmonella* strains to epithelial cells in vitro (Searle et al. 2009; Tzortzis et al. 2005). Moreover, GOS not only reduced the adherence of enteropathogenic *E. coli* microcolonies to HEp-2 and Caco-2 cells but also have the greatest ability to reduce the number of bacteria per microcolony when compared to other oligosaccharides such as fructo-oligosaccharides, inulin, lactulose and raffinose (Shoaf et al. 2006). These findings suggest that GOS may be targeted to the virulence factor that is responsible for microcolony formation.

Plant-derived oligosaccharides, such as pectic- and xylo-oligosaccharides can also inhibit pathogen infection through direct interaction mechanisms. Pectic oligosaccharides (POS) have demonstrated antiadhesion properties against the Shiga toxin-producing *E. coli* O157:H7 in human colon adenocarcinoma epithelial cells (HT29). Di et al. (2017) recently reported that oligo-galacturonic acids (low molecular weight de-esterified structures) are responsible for Shiga toxin-producing *E. coli* antiadhesive activity. Ebersbach et al. (2012) concluded that xylo-oligosaccharides (DP 2–6) significantly decreased the ability of *L. monocytogenes* to adhere to Caco-2 cells, as well as by negatively affecting the expression of adhesion-related genes of the pathogen. Cranberry xyloglucans with SSGG (arabinose–xylose sidechains) have been reported to inhibit *E. coli* O157:H7 adhesion to HT29 cells as well as *E. coli* 1161 to T24 cells, indicating cranberry xyloglucan potential to prevent urinary tract infections as well as gastrointestinal illnesses (Hotchkiss et al. 2012). Other antiadhesive oligosaccharides include oligofructose and inulin, which have been shown to protect mice from systemic infection with *Listeria monocytogenes* and *Salmonella typhimurium* (Buddington et al. 2002). *Listeria monocytogenes* pathogenicity was repressed by cellobiose through downregulation of its virulence factors (Park and Kroll 1993). Inulin also reduced the incidence of traveller’s diarrhoea (Cummings et al. 2001), via stimulation of the immune system through macrophage activation (Meyer et al. 2000). The mechanisms for these structurally diverse oligosaccharides to inhibit bacterial adhesion, toxin binding to receptors or bacterial invasion are unclear since receptor mimicry is unlikely (Rhoades et al. 2008). Although substantial evidence suggesting the protective effect of prebiotics exists, further in vitro and in vivo studies are required to establish structure–function relationships and elucidate certain preventive bioactivity effects of prebiotic oligosaccharides against gastrointestinal infections.

## Adhesion-related immunomodulation of probiotics

Although the relationship between adhesion ability of probiotics and immunomodulation remains unclear, the adherence of some probiotic bacteria, at least temporary, to the gastrointestinal mucosa might be necessary to stimulate the host’s immune system. Probiotics can interact with pattern recognition receptors (PRRs) such as Toll-like receptors (TLRs) of dendritic cells and macrophages through microbe-associated molecular patterns (MAMPs) that are present in the bacterial cell surface or that can be secreted in the environment. This close contact with host immune cells could facilitate surface bound components and other molecules secreted by probiotic in triggering a signalling cascade leading to immunomodulation. An example of MAMPs in some probiotic bacteria are pili structures at its surface. Pili are external molecules of bacteria that play an essential role in the adhesive ability of bacteria and TLR-2 signalling (Lebeer et al. 2012; Turroni et al. 2013). For instance, the cluster SpaCBA polymeric pili present in some probiotic bacteria such as *L. rhamnosus* GG (LGG) contain a mucus binding adhesin in the tip that would facilitate the adherence to IECs. Lebeer et al. (2012) demonstrated in an in vitro study with Caco-2 cells that SpaCBA pili were key for the adhesion ability of LGG to IECs and that this adherence was needed for immunomodulatory activity. Adhesion of LGG to Caco-2 cells through pili reduced the expression of the proinflammatory cytokine IL-8, induced by other MAMPs of LGG as lipoteichoic acid, compared to a LGG mutant without the pili structures. Moreover, the reduction of IL-8 was more evident with an exopolysaccharide LGG mutant, since the EPS removal lead to of more accessible and exposed surface piliation and consequently higher adhesion ability of LGG. Another recent study in LGG (Vargas García et al. 2015) showed that SpaCBA pili played also an important role in the adherence of LGG to macrophages. This interaction promoted antiinflammatory effects by induction of IL-10 mRNA and reduction of IL-6 mRNA in murine macrophages.

On the other hand, some studies have evidenced the effectiveness of probiotics in the treatment and/or prevention of immune associated diseases such as inflammatory bowel diseases (IBDs) (Matsuoka et al. 2018; Tamaki et al. 2016; Peng et al. 2019) An elevated production of the potent inflammatory interleukin (IL)-17 by T-helper (Th)17 cells and other producing cells seems to be involved in the pathogenesis of these diseases (Ueno et al. 2018). However, the role of Th17 cells in health and disease is poorly understood. Although the presence of Th17 cells during steady state (homeostatic condition) promotes the epithelial barrier function, they also could initiate a pro-inflammatory immune response during infection (Stockinger and Omenetti 2017) and turn into pathogenic Th17 cells causing the development of autoimmune and inflammatory conditions. Although mechanisms are still unclear, some studies in rodent models have revealed that the adhesion of some specific commensal bacteria could induce Th17 accumulation. For instant, a recent work conducted by Atharashi and colleagues (Atarashi et al. 2015) in mice demonstrated a strong correlation between epithelial adhesion by enteropathogenic *E. coli* and segmented filamentous bacteria (SFB) and Th17 induction. Moreover, the same study showed that commensal bacterial strains from human faecal samples of ulcerative colitis (UC) patients that showed adhesive properties to epithelial cells similar to SFB were also able to induce Th17 cells in the mouse colon. Conversely, probiotic bacteria could suppress pathogenic Th17 cells and induce steady-state Th17 cells (Lenoir et al. 2016). For instance, in a recent study conducted by Pagnini and co-workers (Pagnini et al. 2018), the antiinflammatory effects and adhesive properties of LGG in an ex vivo and in vivo trial with UC patients was demonstrated. After 7 days of treatment, LGG was detectable in the biopsies performed in proximal and distal colon being the probiotic levels higher in the samples of patients who consumed double doses of probiotic. In this regard, a significant reduction of pro-inflammatory cytokines such as tumour necrosis factor alpha (TNF-α) and interleukin (IL-17) was detected in patients who received double doses of probiotic compared to regular doses. A possible explanation for the protective role of probiotics could be the different amounts and types of MAMPs between beneficial and commensal/pathogenic bacteria that could result in different interactions with the intestinal cells and, therefore, trigger different immunological pathways. In this line, a genomic study (Deutsch et al. 2017) on probiotic species of *Propionibacterium freudenreichii* with varied antiinflammatory properties evidenced that the antiinflammatory phenotype could be correlated with the presence of a combination of different surface proteins involved in adhesion and cytoplasmic factors. Future studies should be aimed to elucidate the mechanisms by which beneficial and probiotic bacteria, but no other commensal or pathogenic bacteria, are able to contribute in the immune homeostasis. Identification of surface proteins and their correlation with antiinflammatory properties of strains could lead into the development of new screening strategies to select specific probiotics as therapies for inflammatory diseases.

## Conclusion

Research findings suggest that mechanisms of action of probiotic bacteria are multifactorial involving stimulation and modulation of the immune system, antagonistic effect against pathogens, transient gut colonisation and metabolites secretion. As pathogenesis involves the adherence to epithelial cells, colonisation of the surface and production of effective concentration of toxins, it is reasonable to think that adhesion ability of probiotic bacteria, at least transiently, could exert their beneficial action with similar mechanisms.

During the last decades, numerous in vitro screening studies using cell lines have been conducted to assess the adhesion ability and antagonism against pathogens of multiple probiotic strains. However, the lack of correlation in many cases between in vitro and in vivo studies reflects the need to (1) develop more complex in vitro models to predict mechanisms of action by probiotic bacteria in host-microbiome ecosystems and (2) conduct more well-controlled and well-designed clinical trials to proof the probiotic efficacy on human health.

Additionally, a better understanding of structure–function relationship is required to elucidate certain preventive bioactivity effects of prebiotic oligosaccharides. Such knowledge could inform clinical studies that would aim at nutritional innervations with probiotics and prebiotics, by thoroughly investigating the optimal dosage, treatment duration for targeted modulation of the gastrointestinal tract and the immune system.
